# SARS‐CoV‐2 infection rate in Antananarivo frontline health care workers, Madagascar

**DOI:** 10.1111/irv.13022

**Published:** 2022-06-26

**Authors:** Rila Ratovoson, Mihaja Raberahona, Rado Razafimahatratra, Lova Randriamanantsoa, Emmanuel Harizaka Andriamasy, Perlinot Herindrainy, Norosoa Razanajatovo, Soa Fy Andriamandimby, Andoniaina Rakotonaivo, Fanirisoa Randrianarisaona, Philippe Dussart, Jean Michel Heraud, Mamy Jean de Dieu de Randria, Matthieu Schoenhals, Rindra Vatosoa Randremanana

**Affiliations:** ^1^ Institut Pasteur de Madagascar Antananarivo Madagascar; ^2^ Centre Hospitalier Universitaire Joseph Raseta Befelatanana Antananarivo Madagascar; ^3^ Centre Hospitalier Universitaire Anosiala‐Alakamisy Antananarivo Madagascar; ^4^ Centre Hospitalier Manara‐penitra Andohatapenaka Antananarivo Madagascar; ^5^ Present address: Infectious Disease Detection and Surveillance Antananarivo Madagascar; ^6^ Present address: Institut Pasteur de Dakar Dakar Senegal

**Keywords:** COVID‐19, health care worker, infection, Madagascar, sub‐Saharan Africa

## Abstract

**Background:**

Health care workers (HCWs) represent a vulnerable population during epidemic periods. Our cohort study aimed to estimate the risk of infection and associated factors among HCWs during the first wave of severe acute respiratory syndrome coronavirus 2 (SARS‐CoV‐2) in Madagascar.

**Methods:**

A prospective cohort study was carried out in three hospitals that oversaw the first cases of COVID‐19. Monthly ELISA‐based serological tests were conducted, and nasopharyngeal swabs were collected in the case of symptoms linked to COVID‐19 for RT–PCR analysis. Survival analyses were used to determine factors associated with SARS‐CoV‐2 infection.

**Results:**

The study lasted 7 months from May 2020. We included 122 HCWs, 61.5% of whom were women. The median age was 31.9 years (IQR: 26.4–42.3). In total, 42 (34.4%) had SARS‐CoV‐2 infections, of which 20 were asymptomatic (47.6%). The incidence of SARS‐CoV‐2 infection was 9.3% (95% CI [6.5–13.2]) person‐months. Sixty‐five HCWs presented symptoms, of which 19 were positive by RT–PCR. When adjusted for exposure to deceased cases, infection was more frequent in HCWs younger than 30 years of age (RR = 4.9, 95% CI [1.4–17.2]).

**Conclusion:**

Our results indicate a high incidence of infection with SARS‐CoV‐2 among HCWs, with a high proportion of asymptomatic cases. Young HCWs are more likely to be at risk than others. Greater awareness among young people is necessary to reduce the threat of infection among HCWs.

## 1 INTRODUCTION

Pandemics have occurred throughout history and appear to be increasing in frequency, particularly because of the increasing emergence of viral zoonotic diseases. These are large‐scale outbreaks of infectious diseases that can greatly increase morbidity and mortality worldwide and cause significant economic, social, and political disruption.^1^ Furthermore, the impact on the health care system remains a great challenge, as recently demonstrated through the global spread of severe acute respiratory syndrome coronavirus 2 (SARS‐CoV‐2), which started in December 2019 in Wuhan, Hubei Province, China.^2^

During the pandemic of coronavirus disease 2019 (COVID‐19) caused by SARS‐CoV‐2, health care workers (HCWs) were and are still on the frontlines battling the disease and are the most at risk of acquiring the infection as they are exposed to infected patients. Previous experiences of a similar disease, severe acute respiratory syndrome (SARS), have left a distressing toll on care workers.^3^ However, risk factors for SARS‐CoV‐2 infection among HCWs have not been well described, especially in African countries.^4^ Currently, the COVID‐19 pandemic continues its progression in Africa, which is determined largely by geography and the subsequent availability of resources.^5^ Thus, it is imperative to ensure the safety of HCWs not only to safeguard continuous patient care but also to ensure that they do not transmit the virus.^6^ The main issue in the care setting is to ward off and prevent the spread of COVID‐19 to hospital staff. Moreover, estimating infection rates and risk factors in this population is important to guide public health measures to protect health care workers and their close contacts, ensure the continuity of health care services, and control rates of secondary transmission in the population.

**FIGURE 1:**
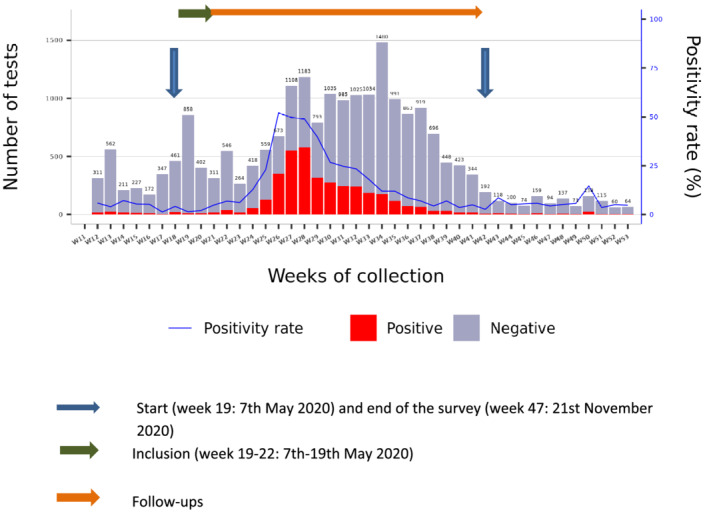
Weekly distribution of the number of tests and the positivity rate in Antananarivo Renivohitra and surrounding (Andramasina, Ambohidratrimo, Antananarivo‐Avaradrano and Antananarivo‐Atsimondrano districts) from the start of COVID‐19 to December 2020 and the correlation to the survey period of HCWs in the three hospitals in Antananarivo (source: Randremanana et al.^7^)

The SARS‐CoV‐2 pandemic hit Africa on 25 February 2020 and Antananarivo, Madagascar on 19 March^7^; a health emergency was declared on 21 March. Despite restrictions implemented to slow the spread of the outbreak, Madagascar experienced the epidemic in different waves and locations. The first city affected was Toamasina, located on the East coast, in April–June 2020. Antananarivo, the capital city with around 2.6 million inhabitants, experienced its first wave between June and August 2020. Specimens obtained from five health districts located in and around the capital (Andramasina, Ambohidratrimo, Antananarivo‐Avaradrano, Antananarivo‐Atsimondrano, and Antananarivo‐Renivohitra districts) and analysed in the Virology Unit at the Pasteur Institute of Madagascar showed that the epidemic started in Antananarivo on the week 24 (8–14 June), it peaked on the week 28 (6–12 July) with the positivity rate of 50% (Figure 1).^7^

For the first month, the number of new daily COVID‐19 cases was relatively low, but this number increased drastically after 42–46 days with an exponential trend and has certainly increased the risk of infection among HCWs in relation to worldwide pressure to produce enough barrier material (masks, hydroalcoholic gel, etc.) and personal protective equipment (PPE). Moreover, while the general population was advised to stay at home to adhere to social distancing rules, HCWs had to go to work in hospitals. Furthermore, the risk for infection may have been highest at the beginning when HCWs were not familiar with the use of PPE.^8^

The World Health Organization (WHO) recommends the implementation of studies targeting HCWs assigned to care for patients with COVID‐19, aiming to assess the risk of infection and transmission in this population.^9^, ^10^ Although many serological surveys have been performed in high‐ and middle‐income countries assessing the risk for SARS‐CoV‐2 infection and seroconversion among frontline health care personnel,^11^, ^12^, ^13^ few studies have been published in sub‐Saharan countries.^14^, ^15^, ^16^ The Pasteur Network in Africa conducted a multicentre prospective study entitled ‘COVID‐19 evaluation risk among health care workers in Africa’ (‘COVER‐HCW’), based on one of the WHO's master protocols.^17^ Such studies are crucial to inform decision‐makers about better control strategies for HCWs in Africa and represent an opportunity to study infections in asymptomatic or paucisymptomatic persons. Madagascar was among the countries that participated in the study during the first wave through the main hospitals that oversaw the first cases of COVID‐19 in Antananarivo.

The aim of the present work was to assess SARS‐CoV‐2 risk infection among frontline COVID‐19 HCWs during the first wave of SARS‐CoV‐2 in Antananarivo and explore risk factors for infection.

## 2 METHODS

### 2.1 Study site

**FIGURE 2:**
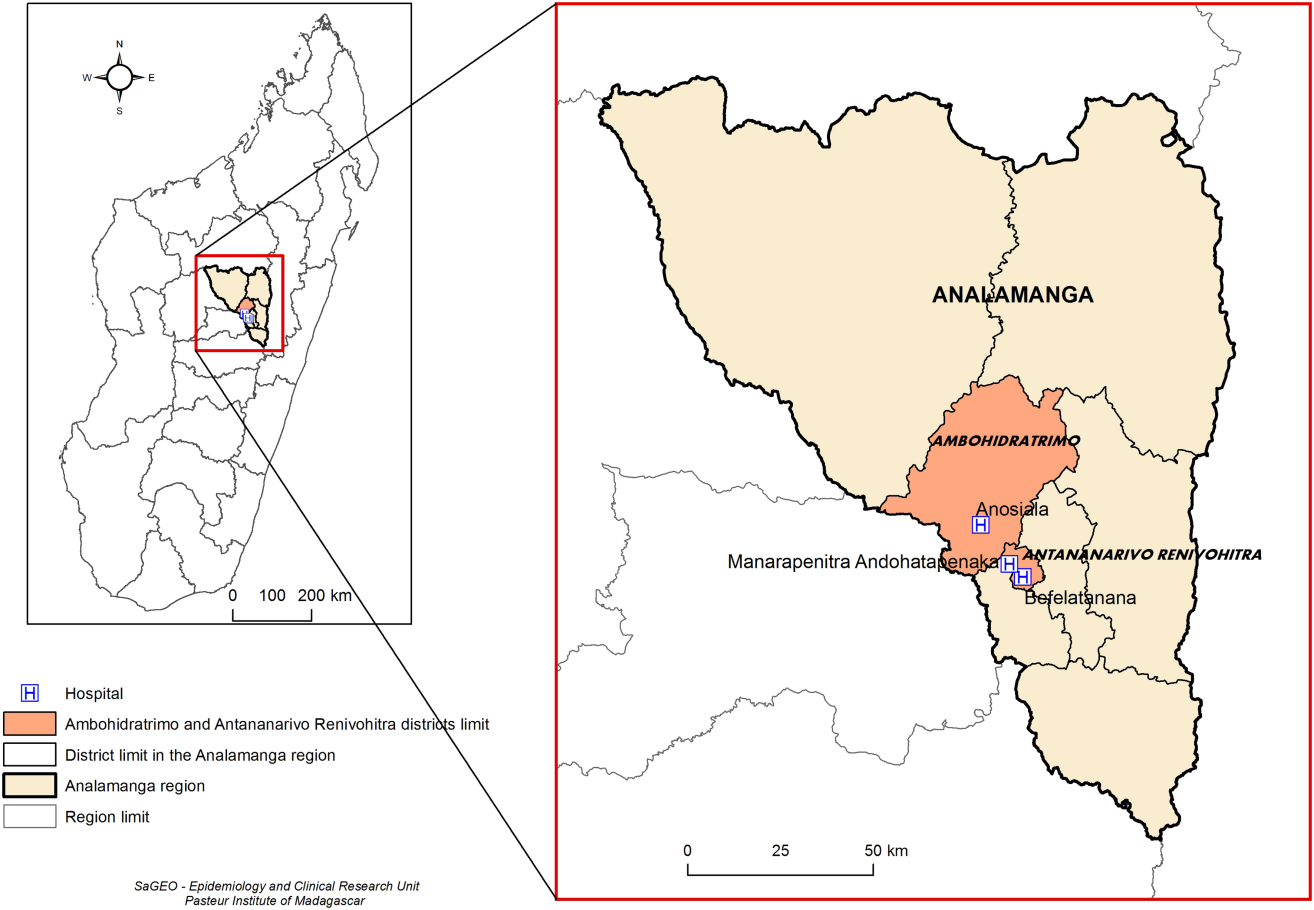
Map of Madagascar including Analamanga region and the districts that include the three hospitals involved in the survey of HCWs in Antananarivo, Madagascar (May–October 2020)

At the beginning of the outbreak, the first confirmed cases were isolated and treated at one of the three main hospitals of Antananarivo (Befelatanana Hospital, Anosiala Hospital and Andohatapenaka Hospital), regardless of the presence of symptoms. The hospitals of Befelatanana and Andohatapenaka are located in the centre of the city (Antananarivo Renivohitra district) while Anosiala is located in the western suburb (Ambohidratrimo) of Analamanga region (Figure 2).

The study was conducted in the wards where suspected SARS‐CoV‐2 patients were managed and confirmed cases were treated. The frontline staff in the three hospitals consisted mainly of physicians and paramedics working in the reception, triage and emergency departments, the infectious diseases department and the medical and paediatric departments. Nursing assistants, physiotherapists, stretcher bearers, radiologists and anaesthesiologists were also involved. As the number of cases increased, in addition to the workload, the other departments received also patients infected with COVID‐19.

### 2.2 Participants and data collection

During May 2020, all frontline staff were invited to participate in the cohort study on a voluntary basis. At the beginning of the study, in each hospital, after obtaining the director's agreement, all HCWs involved in the care of Covid‐19 patients were gathered for an information session on the project.

At inclusion, participants were asked to complete a questionnaire with medical history, current symptoms and compliance with information on infection prevention and control measures. We also administered a questionnaire to each hospital director to review infection control and prevention protocols in health care facilities.

During follow‐up, each participant was asked to complete a symptom diary to record the presence or absence of various signs each day over 15 days. The investigators collected the symptom diary the following month. In case of suspected COVID‐19 symptoms, participants contacted the investigators for nasopharyngeal or oropharyngeal specimen sampling (NP/OP) that were sent to the virology unit at the Institut Pasteur de Madagascar, where they were tested for SARS‐CoV‐2 using real‐time RT–PCR (RT–qPCR) as previously published.^7^ Investigators collected monthly blood samples for serological testing, which was a semiquantitative indirect ELISA for the detection of immunoglobulin G (IgG) for SARS‐CoV‐2 (ID Screen® SARS‐CoV‐2‐N IgG Indirect ELISA Kit, ID.vet, Grabels, France) and a qualitative ELISA for the detection of total antibodies (including IgM and IgG) for SARS‐CoV‐2 (WANTAI SARS‐CoV‐2 Ab ELISA, Beijing Wantai Biological Pharmacy Enterprise Co., Beijing, China) provided by the WHO and previously described.^18^ The ELISA tests carried out in the Immunology of Infectious Diseases Unit at the Pasteur Institute of Madagascar were conducted according to the manufacturer's instructions. According to the manufacturers, the ID Screen® SARS‐CoV‐2‐N IgG Indirect ELISA has a specificity of 99.8% (95% CI 99.3–99.9) and a sensitivity of 95.2% (95% CI 95.2–100),^19^ and the WANTAI kit has a specificity of 100% and a sensitivity of 94.36%.

Exposures to patients infected with SARS‐CoV‐2 were also studied. For HCWs who were managing patients with severe forms of COVID‐19, a form was completed in case of death or discharge of an admitted infected patient. This information was collected throughout the study period.

A SARS‐CoV‐2‐infected HCW was defined as a person either with laboratory confirmation of SARS‐CoV‐2 infection (RT–qPCR) or with seroconversion at any time point; the result of these tests was irrespective of clinical symptoms. Seroconversion was defined as a person who had a serum specimen that tested negative at inclusion and became positive by either of the abovementioned tests during the follow‐up period.

### 2.3 Statistical analysis

Descriptive statistical analysis was used to summarize the characteristics of HCWs; chi‐square tests were performed for categorical variables and survival analysis to examine the relation between infection and HCW characteristics. For survival analysis, the duration of the follow‐up time lasted from the date of inclusion until the date of positivity, either by RT–qPCR or by serology for those who became positive; it was right‐censored for those who did not present the event, and it ended at the date of last report for those who were not followed up until the end. HCWs who had been positive at inclusion, either by RT–qPCR or serology, and those who did not have complete follow‐up were excluded from the Cox model analysis. A backward stepwise selection variable (less than 0.20) was used in the univariate analyses to choose the final model in the multivariate analysis. All *p* values < 0.05 were considered statistically significant. All analyses were conducted with R software.^20^

## 3 RESULTS

### 3.1 Characteristics of HCWs

The study lasted 7 months, with the start of inclusion on 7 May 2020 (week 19) and 5 monthly follow‐ups. The inclusion period lasted 3 weeks, and the last follow‐up, which started in October, ended in November 2020 (week 47) (Figure 1). We invited 337 HCWs, and 122 (36.2%) agreed to participate in the study, of which 18 (15%) were from Anosiala Hospital, 49 (40%) from Andohatapenaka Hospital and 55 (45%) from Befelatanana Hospital. Among the participants, 61.5% (75/122) were female. The majority of HCWs who participated in the study were physicians (34.4%), trainee physicians (29.5%), paramedical staff (23.8%), and the others (physiotherapists, laboratory personnel, stretcher‐bearers, etc.) made up 12.3%. Most were middle‐aged adults with a median age of 31.9 years (interquartile range IQR: [26.4–42.3 years]), and 28.7% (35/122) of participants reported having at least one comorbidity (hypertension, heart disease, diabetes, obesity, asthma or other lung disease) (Table 1).

**TABLE 1:** Characteristics of the 122 HCW participants according to infection status from inclusion to the end of follow‐up (May–October 2020) in three hospitals of Antananarivo, Madagascar

Characteristics	SARS‐CoV‐2‐non‐infected HCWs n = 80		SARS‐CoV‐2‐infected HCWs n = 42		Total n = 122		*p*
Age (median, IQR)	34.2	26.9–45.4	27.6	25.2–36.6	31.9	26.4–42.3	
Age group							**0.01**
30 years and older	51	63.7%	16	38.1%	67	54.9%	
Less than 30 years old	29	36.2%	26	61.9%	55	45.1%	
Gender							0.6
Female	51	63.7%	24	57.1%	75	61.5%	
Male	29	36.3%	18	42.9%	47	38.5%	
Comorbidities							0.5
No comorbidity	55	68.8%	32	76.2%	87	71.3%	
At least one	25	31.2%	10	23.8%	35	28.7%	
Hospital							0.7
Anosiala	12	15.0%	6	14.3%	18	14.8%	
Befelatanana	38	47.5%	17	40.5%	55	45.1%	
Andohatapenaka	30	37.5%	19	45.2%	49	40.2%	
Occupation							0.7
Physicians	30	37.5%	12	28.6%	42	34.4%	
Students and hospital interns	23	28.7%	13	30.9%	36	29.5%	
Paramedical staff	17	21.2%	12	28.6%	29	23.8%	
Others	10	12.5%	5	11.9%	15	12.3%	
Symptomatic during the survey							0.99
No	37	46.2%	20	47.6%	57	46.7%	
Yes	43	53.8%	22	52.4%	65	53.3%	

**FIGURE 3:**
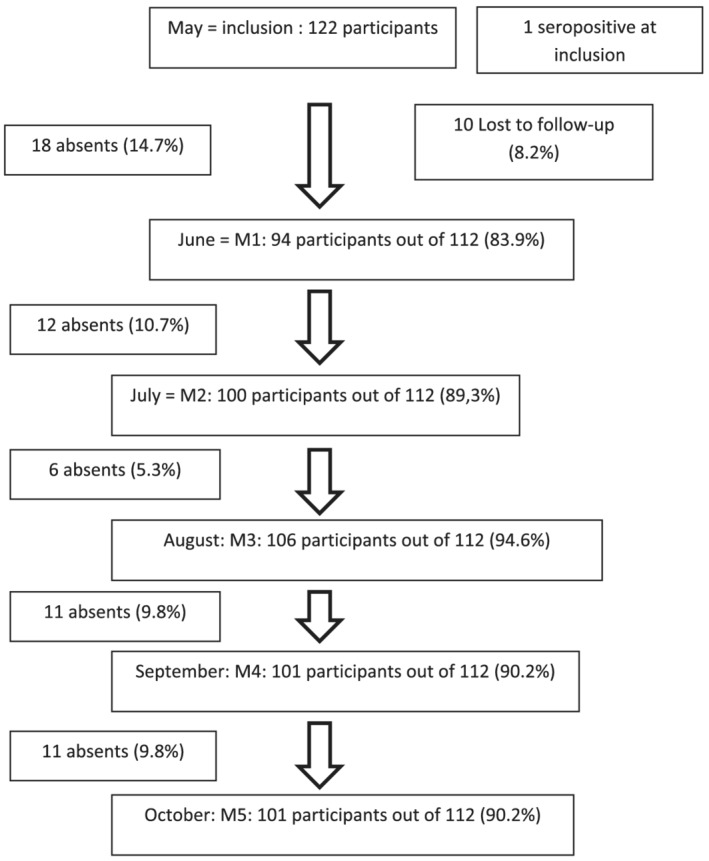
Flow chart of the HCW participants during the survey period (May–October 2020) in the three hospitals of Antananarivo, Madagascar

Of the 122 HCWs, one had already had a positive serology at inclusion and was excluded from the follow‐up data, and 10 were lost to follow‐up after inclusion and did not participate in the monthly follow‐ups. Among five HCWs who presented symptoms (symptomatic HCWs) at inclusion, none tested positive by PCR. At each follow‐up, the proportion of HCWs who participated ranged from 84% to 94% (Figure 3). Of the 112 remaining HCWs who participated in at least one follow‐up visit, 82 HCWs gave valid blood samples from inclusion to the fifth follow‐up and had a complete follow‐up visit during the study. The median age of those who completed the follow‐up visit was 29.6 years old (IQR: [26.0–39.8 years]). For that reason, the cut‐off of to differentiate age groups was 30 years old.

### 3.2 Infection with SARS‐CoV‐2

On the basis of the different serological results, excluding the HCW who had a positive serology at inclusion, 40 seroconverted during the follow‐up, giving a seroprevalence of 36% (40/111). In total, 42 HCWs had evidence of SARS‐CoV‐2 infection at any given time point, detected either by serology or RT–qPCR. Among them, 47.6% (20/42) did not report any symptoms (Table 1). During the study period, 121 NP/OP specimens were sampled from 65 symptomatic HCWs, of which 19 tested positive by RT–qPCR and three tested negative by RT–qPCR but had seroconverted. Ten symptomatic HCWs had three or more NP/OP specimens sampled from inclusion to the end of follow‐up. Among the 19 positive cases detected by RT–qPCR, 18 also had positive serological results.

Among the 65 HCWs with symptoms, eight presented convulsions or unconsciousness, and four had dyspnoea and asthenia or malaise. None were hospitalized or died during the survey period.

**FIGURE 4:**
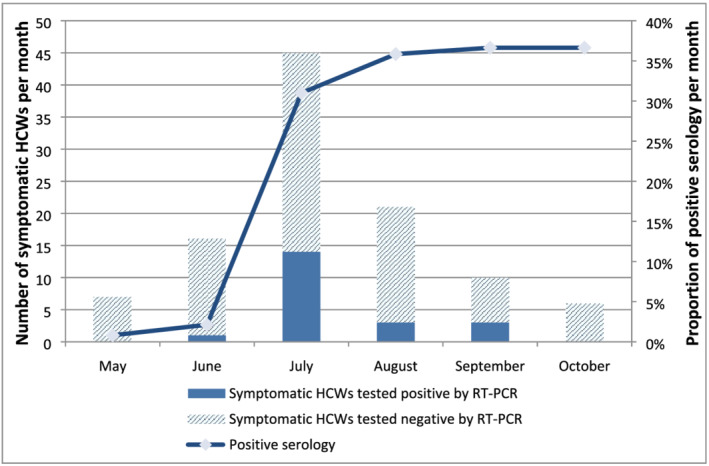
Monthly anti‐SARS‐CoV‐2 seropositivity in HCWs participating in the survey in three hospitals of Antananarivo, Madagascar from May to October 2020

**FIGURE 5:**
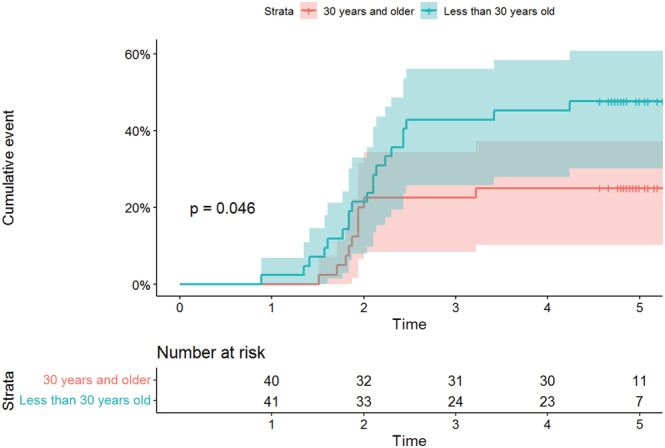
Cumulative event (incidence of infection) by age group of the HCW participants in the survey in three hospitals of Antananarivo, Madagascar (May–October 2020)

Regardless of absences during follow‐ups, HCW seropositivity during the 4 months of post inclusion visits progressed from 2.1% (2/94), 31% (31/100) and 35.8% (38/106) to 36.6% (37/101) and remained stable during the fifth month at 36.6% (37/101) (Figure 4). Survival analysis was performed with 82 individuals who completed all follow‐up visits, corresponding to 320.4 person‐months. Among the 82 HCWs, 30 HCWs had evidence of SARS‐CoV‐2 infection, giving an estimated incidence of 9.4% person‐months (95% CI = [6.5%–13.2%]). In the survival analysis, young HCWs aged younger than 30 years had a higher risk of being infected than others (Table 2). The incidence of SARS‐CoV‐2 infection in HCWs aged younger than 30 years was 13.1% person‐months (95% CI = [8.4%–19.7%]) compared with 5.9% (95% CI = [3.1%–10.9%]) among those aged 30 years and older. The cumulative event (incidence of infection) by age group is shown in Figure 5. In contrast, gender, comorbidities, hospital, occupation and presence or absence of symptoms were not associated with the risk of being infected with SARS‐CoV‐2.

**TABLE 2:** Result of survival analysis in HCWs in three hospitals in Antananarivo, Madagascar

Characteristics	N = 82	SARS‐CoV‐2‐infected HCWs n = 30	Relative risk (RR) 95% CI	*p*	Adjusted RR 95% CI	*p*
Age group				**0.05**		**0.01**
30 years and older	40	10	1		1	
Less than 30 years old	42	20	**2.1 (0.9–4.5)**		4.9 (1.4–17.2)	
Gender				0.4		
Female	47	15	0.7 (0.3–1.5)			
Male	35	15	1			
Comorbidities				0.8		
No comorbidity	59	22	1			
At least one	23	8	0.5 (0.4–2)			
Hospital				0.5		
Anosiala	9	2	1			
Befelatanana	46	15	1.7 (0.4–7.4)			
Andohatapenaka	27	13	2.3 (0.5–10.3)			
Occupation				0.8		
Physicians	25	9	1			
Students and hospital interns	29	10	0.9 (0.4–2.2)			
Paramedical staff	18	8	1.3 (0.5–3.4)			
Others	10	3	0.7 (0.2–2.6)			
Symptomatic during the survey				0.9		
No	34	13	1			
Yes	48	17	0.9 (0.5–1.9)			
Exposure to infected patients who died at hospital^a^				0.06		0.12
No contact with deceased	14	1	1		1	
At least 1 exposure to deceased patient	51	18	5.7 (0.7–42.6)		4.8 (0.6–35.9)	

^a^ Responses from 65 participants.

Regarding exposure to infected patients, 96 HCWs reported having been exposed to positive patients infected with SARS‐CoV‐2 who were cured and discharged, deceased, or other (transferred, etc.). However, considering loss to follow‐up during the survey period and non‐responses from some HCWs, 88 HCWs had information on exposure to infected and deceased patients, and only 65 among the 82 who completed the follow‐ups had that information (Table 2). HCWs had a median of two exposures to deceased patients during the survey period (IQR = [0–3]). Compared with those who had no contact with a deceased patient, having at least one exposure to a deceased patient was associated with a higher risk of infection, but the difference was not statistically significant (RR = 2.6, 95% CI [0. 8–8.6]) (Table 2). In the multivariate analysis adjusted for exposure to infected patients who died in the hospital, the probability of being infected with SARS‐CoV‐2 was 3.7 times higher (95% CI [1.57–8.75]) in HCWs aged younger than 30 years (Table 2).

### 3.3 Compliance with information on infection prevention and control (IPC) measures

At inclusion, 101 HCWs reported having received training in infection prevention and control within the hospital, of which more than 75% of participants reported having received training in March 2020. The proportion of HCWs who reported always using alcohol‐based products or soap as recommended before touching a patient was 90.1% and after touching a patient, 95.0%. All HCWs had worn masks during their exposure to infected patients, but quantitative data from the type of mask was not registered. The majority of masks used were surgical masks for all HCWs. However, according to the quantity granted by the Ministry of Public Health and other donors, FFP2 and N95 masks were also used, especially by doctors and nurses.

Regarding IPC in the three hospitals, one hospital reported having an IPC program, two hospitals had IPC guidelines and an IPC team, and all three hospitals had a dedicated and trained infection preventionist. All three hospitals had IPC guidelines for standard and supplementary (transmission‐based) precautions, and two had regular IPC training for health care workers (at least once a year). At the time of the survey, all three facilities reported having PPE, but only one indicated having it in sufficient quantities. Two hospitals reported that the PPE available was of good quality and appropriate for their needs. Alcohol‐based hand sanitizer was readily available for hand hygiene in one hospital, and soap and water were used in all three. Two hospitals conducted regular hand hygiene audits and provided feedback to HCW staff; other IPC audits were conducted by all three facilities. Two hospitals performed nosocomial infection surveillance for patients, but none for HCWs. Two hospitals always alerted HCWs if a patient infected with SARS‐CoV‐2 was being treated there; for the third hospital, it alerted HCWs only sometimes because their managers were not notified in time.

## 4 DISCUSSION

We conducted this cohort study to estimate the risk of SARS‐CoV‐2 infection and the associated factors among HCWs at the three main hospitals of Antananarivo who cared for COVID‐19 patients. We found that 42 HCWs (34.4%) had evidence of SARS‐CoV‐2 infection at any time point detected either by serology or RT–qPCR; among them, 47.6% (20/42) did not report any symptoms. During the study period, we collected NP/OP specimens from 65 symptomatic HCWs, of which 19 were positive by RT–qPCR. Statistical analysis performed with 82 HCWs who completed all the follow‐up visits showed that the incidence of SARS‐CoV‐2 infection was 9.4% person‐months, and young HCWs aged younger than 30 years had a higher risk of being infected than others.

We found that 34.4% HCWs had evidence of SARS‐CoV‐2 infection at any time point, and among them, 47.6% (20/42) were asymptomatic. Our seroprevalence estimated at 36.0% was much higher than that reported in a recent meta‐analysis of the seroprevalence of SARS‐CoV‐2 antibodies in HCWs in many countries in 2021, with an overall seroprevalence of approximately 8–9%.^11^

Our study is among the few that have been performed in HCWs in sub‐Saharan Africa, and the estimated seroprevalence reported here was higher than those found in urban Malawi, which observed a seroprevalence of 12.5%,^15^ and was quite similar to the seroprevalence reported in the Democratic Republic of Congo at 41.2%.^14^ These observed differences might be explained by differences in adherence to IPC measures, the use of PPE among HCWs, and other measures applied in the different settings (hand hygiene audits, availability of alcohol‐based hand sanitizer, presence of surveillance system for nosocomial infections in patients). We also cannot exclude some differences due to methods, particularly serological tests. We found that the seropositivity in HCWs started to increase since the beginning of the study (May), reaching 31% in July, a trend that seems to parallel that of the epidemic in the general population until July. Then, the proportion of HCWs who had a positive serology reached a plateau (36%) during the last 2 months of the follow‐up period (September to October), as opposed to the number of COVID‐19 cases in the general population, which progressively decreased. This seropositivity plateau might correspond to the persistence of anti‐SARS‐CoV‐2 antibodies after infection with SARS‐CoV‐2.

We found that the pandemic affected young HCWs more than others. The relative risk in HCWs aged younger than 30 years was five times higher than among older HCWs, even when adjusted for the number of contacts with severe disease leading to death. Young people might have behaviours that may lead them to be more at risk at work than others in the community (for example, less precautions taken outside work and during their occupational activities). This might also be explained by the measures adopted by the government, who authorized workers with comorbidities (high blood pressure, diabetes, etc.) to abstain from working or telecommuting; most of them were of advanced age and did not work during a certain period of the pandemic. In the study hospitals, those with concurrent conditions or older individuals who continued to work were asked by their supervisors to not take part in the management of COVID‐19 patients. Thus, it can be assumed that young HCWs had more contact with COVID‐19 patients, especially those with severe disease. The young (median age: 31.9 years) and healthy (68.8% without comorbidities) characteristics of the participants could also explain the absence of hospitalization and death in our cohort. Furthermore, a modelling study indicated that the youthfulness of the African population compared to other continents may have an impact on the dynamics of epidemics, resulting in widespread and mostly asymptomatic infections.^21^ Considering the correlation between the peaks of SARS‐CoV‐2 seroprevalence in the general population and our cohort, we cannot rule out the possibility of contamination of HCWs linked to community exposure. In addition, SARS‐CoV‐2 seroprevalence among blood donors in Antananarivo was 43% at the end of the first wave, confirming the circulation of the virus in the city^18^ This is a population that may not be representative of the general population as they are excluded from giving blood if they have not been healthy for at least a few weeks, implying that seroprevalence in the general population may be higher and could contribute to the contamination of HCWs.

Unlike other studies,^22^, ^23^ no association was found between job categories and SARS‐CoV‐2 infection, although only one hospital reported having a sufficient supply of PPE during the study period. However, all three hospitals have IPC guidelines for standard and supplementary (transmission‐based) precautions, and the majority of HCWs reported always using alcohol‐based products or soap as recommended before (90.1%) and after caring for patients (95.0%). These findings may highlight the efficacy of PPE when appropriately used and the management of IPC, as seen in a previous study.^24^ However, evaluations of PPE use and IPC were self‐reported, and the results should be interpreted with caution. Studies conducted elsewhere show that HCWs who manage severe or critically ill patients are at higher risk of infection.^25^, ^26^, ^27^, ^28^ We found that compared to those who had no contact with a deceased patient, having at least one exposure to a deceased patient was associated with a higher risk of infection, but the difference was not statistically significant. Our study might have had a lack of power, as we had less information on exposure to infected and deceased patients. Patients with severe forms of disease may have required more aggressive care (procedures that may generate aerosol such as high/low oxygen, invasive ventilation, etc.) or more frequent contact with the nursing staff and physicians during their hospital stay because they needed more meticulous care. The virus can be viable up to 1 month in severe patients versus generally less than 10 days in regular patients.^29^, ^30^, ^31^

Our study had some limitations. Participation in our study was voluntary, and our sample of HCWs comprised those who were willing to take part in the study. This could have affected the incidence and risks found. It is possible that people who felt more at risk (older people, with comorbidities) would have participated. However, we observed that the cohort was young, and only 32% had a comorbidity. Our limited sample size may also explain the absence of statistical significance observed for some of the factors listed by other studies to be potential risk factors for SARS‐CoV‐2 infection among HCWs.

We had difficulties in conducting surveys among HCWs, and only 36% of those invited consented to participate in the study. However, for those who agreed to participate, we observed good compliance with the study. The proportion of HCWs who participated at each follow‐up was high and ranged from 84% to 94%, and 73% had a complete follow‐up visit during the study. One limitation is that we were not able (and it's difficult to do so) to assess whether HCW contamination occurred in the community or at the hospital.

## 5 CONCLUSION

Our study confirmed that HCWs on the front lines are at high risk of contracting SARS‐CoV‐2 infection. No infected participant developed a severe form or was hospitalized, likely contributing to high community transmission. Greater awareness among young HCWs is necessary to reduce the threat of infection. In particular, promoting vaccination of HCWs is an important strategy not only to protect HCWs and reduce absenteeism following infection of staff but also to reduce potential nosocomial infections in hospitalized patients. Other strategies to strengthen IPC, such as regular staff training and PPE provisions, should be continued. There is a high proportion of asymptomatic infections among HCWs, and periodic antigenic screening for the early detection of infections might be useful to assist decision‐makers in the prompt management of infected HCWs.

## CONFLICT OF INTEREST

All the authors have no conflicts of interest to declare.

## AUTHOR CONTRIBUTIONS

**Rila Ratovoson:** Data curation; formal analysis; investigation; supervision; writing‐original draft; writing‐review and editing. **Mihaja Raberahona:** Investigation; supervision; writing‐review and editing. **Rado Razafimahatratra:** Investigation; supervision; writing‐review and editing. **Lova Randriamanantsoa:** Investigation; supervision; writing‐review and editing. **Emmanuel Andriamasy:** Data curation; investigation; supervision; writing‐review and editing. **Perlinot Herindrainy:** Methodology. **Norosoa Razanajatovo:** Data curation; formal analysis; supervision; writing‐review and editing. **Soa‐Fy Andriamandimby:** Data curation; formal analysis; supervision; writing‐review and editing. **Andoniaina Rakotonaivo:** Investigation; writing‐review and editing. **Fanirisoa Randrianarisaona:** Data curation; formal analysis; writing‐review and editing. **Philippe Dussart:** Supervision; validation; visualization; writing‐review and editing. **Jean‐Michel Heraud:** Conceptualization; funding acquisition; methodology; supervision; validation; visualization; writing‐review and editing. **Mamy Randria:** Investigation; project administration; supervision; writing‐review and editing. **Matthieu Schoenhals:** Conceptualization; data curation; methodology; supervision; validation; visualization; writing‐review and editing. **Rindra Randremanana:** Conceptualization; data curation; formal analysis; funding acquisition; methodology; project administration; supervision; validation; visualization.

## Data Availability

The data that support the findings of this study are available from the corresponding author upon reasonable request.
